# On the Influence Exerted by Chloral on the Pain of Parturition

**Published:** 1870-11

**Authors:** 


### On the Influence exerted by Chloral on the Pain of Parturition.

The *Edinburgh Medical Journal*, for August, 1870, contains an interesting article on this subject, by E. Lambert, Esq. The following are the author's conclusions:

1. Chloral is an agent of great value in the relief of pain during parturition.

2. It may be administered under favorable circumstances during, and at the close of the second stage, with the result of producing absolute unconsciousness in the same sense in which we understand unconsciousness under chloroform.

3. When thus given successfully, it has the advantage over chloroform, that it requires no interference with the patient.

4. It is desirable to retain chloroform in the position which it at present occupies in midwifery, and to reserve for the agency of chloral the first stage of labor. If, however, chloral, or some agent having analogous properties, is found successfully to relieve the pain of uterine contraction, the use of chloroform will be restricted to a lesser period of the duration of labor, or to the facilitation of manual or instrumental interference.

5. It is demonstrated that a labor can be conducted, from its commencement to its termination, without any consciousness on the part of the patient, under the sole influence of chloral.

6. The exhibition of chloral in no wise interferes with the exhibition of chloroform.

7. The proper mode of exhibiting chloral is in fractional doses of gr. xx. every quarter of an hour, until some effect is produced; and according to the nature of that effect, the further administration is to be regulated. Some persons will require doses of ʒj; and it is better to produce an anæsthetic effect ʒiij, by giving in the space of two hours, than by ʒj given singly.

8. The effects of chloral are continued beyond the period of completed parturition, and the repose experienced by the patient after her labor, is one of the favorable circumstances to be noted in considering its application to childbirth.

9. Any stimulating effects, in the form of general excitability, occasionally observed during the administration, have passed away very rapidly.

10. Chloral not only does not suspend, but rather promotes uterine contraction, by suspending all reflex actions which tend to counteract the incitability of the centres of organic motion.

11. Labors under chloral will probably be found to be of shorter duration than when natural, for unconscious contractions appear to have more potent effects than those which are accompanied by sensation of pain.

12. Experiments are required in order to determine whether there exists the same antagonism between ergot and chloral, as is known to exist between strychnia and chloral.

13. The general conditions under which chloral is to be administered, are the same as those which regulate the administration of chloroform, and the rules laid down by Sir James Simpson, in connection with this subject, must be rigidly adhered to.—*American Journal of the Medical Sciences*, October, 1870.

